# Synthesis, spectroscopic, electrochemical and photophysical properties of high band gap polymers for potential applications in semi-transparent solar cells

**DOI:** 10.1186/s13065-021-00751-4

**Published:** 2021-04-21

**Authors:** Peshawa O. Amin, Kamal Aziz Ketuly, Salah Raza Saeed, Fahmi F. Muhammadsharif, Mark D. Symes, Avishek Paul, Khaulah Sulaiman

**Affiliations:** 1 Charmo Center for Research, Training and Consultancy, Charmo University, 46023 Chamchamal, Kurdistan Region Iraq; 2 Department of Medical Chemistry, College of Medicine, University of Duhok, Duhok, Kurdistan Region Iraq; 3 Low Dimensional Materials Research Centre (LDMRC), Department of Physics, Faculty of Science, Universiti Malaya, 50603 Kuala Lumpur, Malaysia; 4 Department of Physics, Faculty of Science and Health, Koya University, Koya, KOY45 Kurdistan Region, F.R. Iraq; 5 WestCHEM, School of Chemistry, University of Glasgow, Glasgow, G128QQ Scotland UK

**Keywords:** Conjugated polymers, Synthesis, FTIR, CV measurements, Optoelectronic parameters, Semi-transparent photovoltaics

## Abstract

**Background:**

The design of new polymers able to filter the electromagnetic spectrum and absorb distinctly in the UV and high-energy part of visible spectrum is crucial for the development of semi-transparent solar cells. Herein, we report on the synthesis and spectroscopic, electrochemical, and photophysical characteristics of three new polymers, namely (i) Poly(triamterene-co-terephthalate), (ii) Poly[triamterene-co- 3-(2-pyridyl)-5,6-diphenyl-1,2,4-triazine-p,p′-disulfonamide], and (iii) Poly(5-hydroxyindole-2-carboxylate) that might show promise as materials for semi-transparent solar cells.

**Results:**

The energy band gap, refractive index, dielectric constant, and optical conductivity of the electron donor polymer, poly(triamterene-co-terephthalate), were determined to be 2.92 eV, 1.56, 2.44 and 2.43 × 10^4^ S cm^−1^, respectively. The synthesized electron acceptor polymers showed a relatively high refractive index, dielectric constant, and optical conductivity. The presence of a direct allowed transition was confirmed between intermolecular energy bands of the polymers.

**Conclusions:**

The polymers showed relatively high energy gap and deep HOMO levels, making them strong absorbers of photons in the UV region and high energy part of the visible region. The synthesized donor and acceptors performed well relative to P3HT and fullerenes due to the close match of the HOMO and LUMO levels. With further development, the polymers could be viable for use as the active layers of semi-transparent solar cells.

## Introduction

Polymer solar cells are contributing significantly to the production of large area and cost-effective sources of renewable power with a reduced environmental impact. Despite tremendous efforts in proposing different device architectures such tandem, bilayer, and bulk hetero junction to improve the power conversion efficiency (PCE) of solar cells, synthesis of new polymer materials is of great importance for cultivating and diversifying soft materials’ applications in photovoltaic technology [1–3]. Due to the discrete absorption spectra of organic semiconductors, light harvesting is inadequate whereas this drawback opens a window of opportunity for transparent photovoltaic devices. Based on energy distribution, the solar spectrum is divided into three main regions which are ultraviolet (UV), visible (Vis), and infrared (IR) [4]. For transparent or semi-transparent photovoltaic cells, it is imperative to have an active layer, transport layers and electrodes exhibiting some degrees of transparency in the visible region, while absorbing photon energy in the UV and IR regions [5]. Because of the promising applications of transparent and semi-transparent polymer solar cells in building-Integrated Photovoltaics (BIPV), vehicles, and mobile electronic devices, several studies have employed new strategies for improving power conversion efficiency and visibility. These improvements include device architecture and synthesis of new polymers [6–10]. Moreover, there is a tradeoff between average visible transparency (AVT) and PCE. In addition to enhancing the transparency of the electrodes by reducing the thickness or using Ag nanoparticles and transporting layers, selecting polymers as the active layers is crucial for balancing light harvesting and visibility [11–13]. Therefore, a broad study of new material structures is required for better assessment of photo generation of excitons and their separation at the interface between donor and acceptor materials. The main method for designing a new structure is through restructuring of conjugated polymers by introducing different kinds of functional groups or attached structures like aromatic rings, or by adjusting the side chains [14, 15]. A review of the literature revealed that different types of narrow bandgap polymers have been synthesized to improve intramolecular charge transfer by introducing electron withdrawing groups [15–20]. Interestingly, narrow bandgap materials cover the IR region of the solar spectrum, whereas high bandgap materials cover the UV region. In this paper, three new polymers are synthesized from the precursors triamterene (2,4,7-Triamino-6-phenylpteridine, 6-Phenyl-2,4,7-pteridinetriamine), 3-(2-Pyridyl)-5,6-diphenyl-1,2,4-triazine-*p*,*p*′-disulfonic acid monosodium salt hydrate and 5-Hydroxyindole-2-carboxylic acid, as shown in Table 1. Therefore, in one-step reactions, two electron-rich polymers: (i) Poly (triamterene-co-terephthalate) and (ii) Poly[triamterene-co-3-(2-pyridyl)-5,6-diphenyl-1,2,4-triazine-p,p′-disulfonamide], and one electron-accepting polymer Poly (5-hydroxyindole-2-carboxylate) (iii) were synthesized. The spectroscopic, photophysical and electrochemical characteristics of the new polymers were examined along with a comprehensive study of their energy gaps, HOMO and LUMO levels and their optical constants.

**Table 1:** Synthesized materials, molecular structure, nomenclature and their labels

Precursor	Molecular structure	Synthesized polymer	Label
Triamterene (2,4,7-triamino-6-phenylpteridine, 6-Phenyl-2,4,7-pteridinetriamine)		Poly(triamterene-co-terephthalate)	P(TRI-co-TER)
The 3-(2-pyridyl)-5,6-diphenyl-1,2,4-triazine-p,p’-disulfonic acid, + Triamterene		Poly[triamterene-co- 3-(2-pyridyl)-5,6-diphenyl-1,2,4-triazine-p,p′-disulfonamide]	P(TRI-co-DISULF)
5-Hydroxyindole-2-carboxylic acid		Poly(5-hydroxyindole-2-carboxylate)	PINDOLE

## Materials and methods

### Syntheses of polymers

#### Synthesis of poly(triamterene-co-terephthalate)

**Fig. 1:**
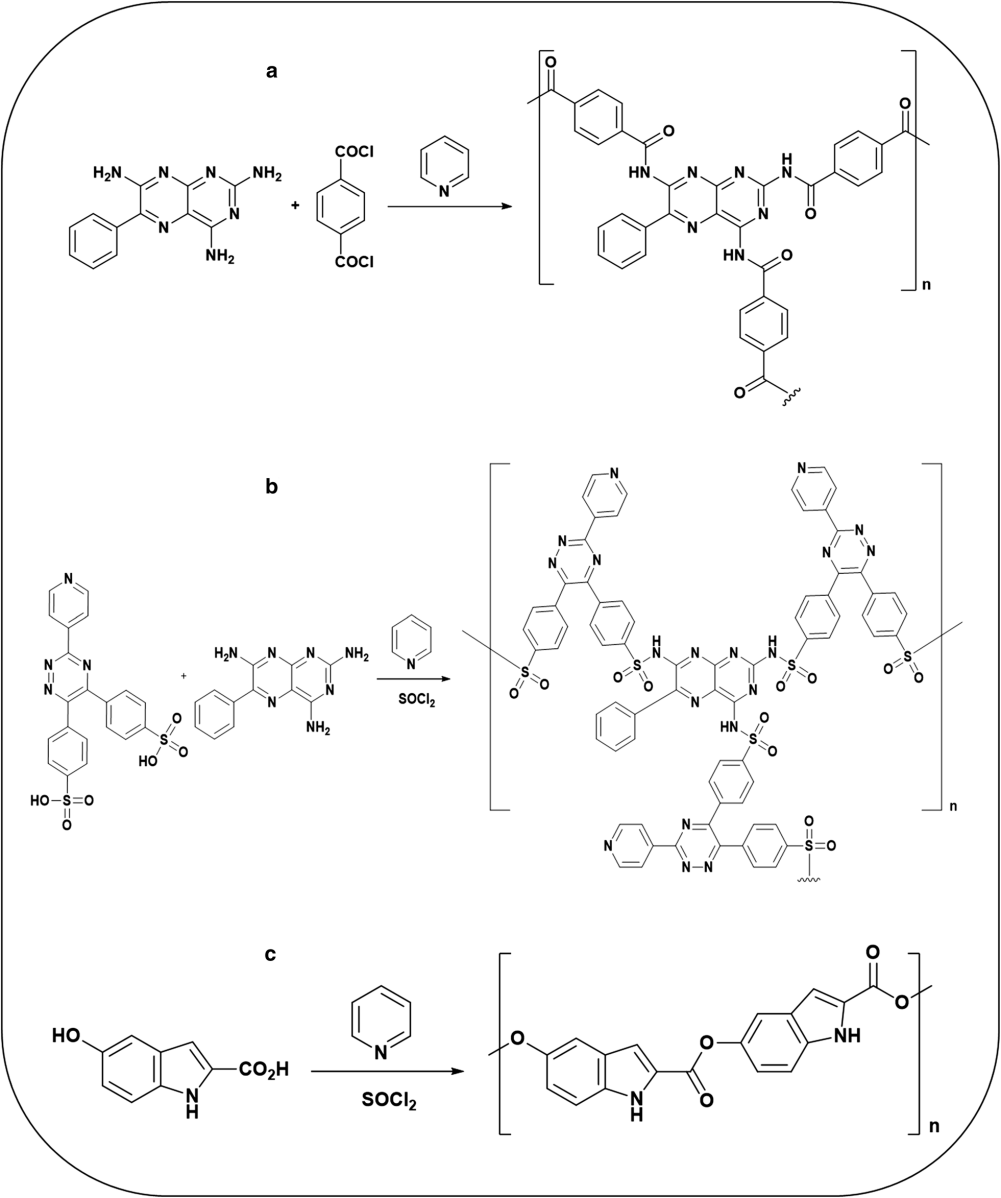
Synthesis route of **a** Poly(triamterene-co-terephthalate), (P(TRI-co-TER)), **b** Poly[triamterene-co- 3-(2-pyridyl)-5,6-diphenyl-1,2,4-triazine-p,p’-disulfonamide], (P(TRI-co-DISULF)), and **c** Poly(5-hydroxyindole-2-carboxylate) (PINDOLE)

Triamterene (2,4,7-triamino-6-phenylpteridine,6-phenyl-2,4,7-pteridinetriamine), 99% (2.35 g) and terephthaloyl chloride flakes, 99% (2.86 g) were added into a conical flask fitted with an air condenser and blue silica drying tube. Pyridine (75 ml) was added and the solution stirred and refluxed on a hot plate for 1 h (Fig. 1a). A deep orange suspension was produced and the solution was left to cool. The reaction mixture was filtered through filter paper under vacuum. The orange residue was washed with *n*-hexane: acetone: ethylacetate (70:10:20, v/v/v, 100 ml). The deep yellow solid product was dried under vacuum at RT and yielded 4.06 g. The ^1^H-NMR spectra showed the 5 phenyl protons in the triamterene: δ 7.42, δ 7.85, δ 8.473 ppm and the 4 aromatic protons in the terephthalate: δ 8.006. The IR spectra gave λ = 1743 cm^−1^ (C=O stretch); 1327 cm^−1^(C–N stretch) and the phenyl 1500, cm^−1^ (C=C stretch), 713 cm^−1^ (C–H bend aromatic).

#### Synthesis of poly[triamterene-co- 3-(2-pyridyl)-5,6-diphenyl-1,2,4-triazine-p,p′-disulfonamide]

The 3-(2-pyridyl)-5,6-diphenyl-1,2,4-triazine-p,p′-disulfonic acid, monosodium salt hydrate, 97% (2.36 g) was added into a 3-necked round bottom flask with magnetic bar and fitted with a condenser and silica gel drying tube. Pyridine (42 ml) was added and the yellow solution was stirred and thionyl chloride (23 ml) was added at RT. Triamterene (2,4,7-triamino-6-phenylpteridine, 6-phenyl-2,4,7-pteridinetriamine), 99% (1.16 g) was added to this solution after 20 min and the solution colour changed from yellow/orange to a dark wine-red during a further 15 min of stirring (Fig. 1b). The drying tube was then removed and temperature increased to 110 °C, until there was no more HCl liberation (2 h). The dark gummy product was suspended in and washed with *n*-hexane, ethyl acetate and chloroform then filtered under vacuum and dried under vacuum, yielding (2.95 g). The ^1^H-NMR spretrum showed the eight aromatic protons of the diphenyl groups: δ 8.005 ppm; the 4 pyridyl protons: δ 8.17, δ 8.28, δ 8.99 & δ 9.3 ppm; the 5 protons of the phenyl of triamterene: δ 8.54, δ 8.77 and the NH proton: δ 8.56. The IR bands were at λ = 3450, 1647 cm^−1^ (N–H stretch), the phenyl group 1571, 1500 cm^−1^ (C=C stretch), 713 cm^−1^ (C–H bend aromatic) and 1371, 1325 cm^−1^ (S=O stretch).

#### Synthesis of poly(5-hydroxyindole-2-carboxylate)

The 5-Hydroxyindole-2-carboxylic acid, 98% (4.98 g) was added into a 3-necked round bottom flask provided with a magnetic stirrer and condenser fitted with a blue silica drying tube. Pyridine (50 ml) was added with stirring and thionyl chloride (10 ml) was added drop wise at RT over 20 min (Fig. 1c). This solution was refluxed (35 min) on the hot plate until there was no more liberation of HCl and left to cool at RT. The gummy dark brown product was suspended in chloroform and filtered under vacuum and dried under vacuum, yielding 4.1 g. ^1^H-NMR spectroscopy showed the aromatic three protons: δ 7.91 (m), δ 8.45 (m), δ 8.71 (d) ppm and the indole, CH proton: δ 7.93 and the NH proton: δ 8.46. The IR bands were at λ = 3379 cm^−1^ (N–H stretch, pyrrole ring); 1665 cm^−1^ (C=O stretch); 1284, 1371 cm^−1^ (C–N stretch) and the aromatic group 1500 cm^−1^ (C=C stretch) and 713 cm^−1^ (C–H bend).

### Materials characterization

The 3-(2-pyridyl)-5,6-diphenyl-1,2,4-triazine-p,p′-disulfonic acid, monosodium salt hydrate, (97%) was from Merck and the rest of the compounds were from Alfa Aesar. The pyridine was dried over sodium hydroxide pellets and distilled from it. The ^1^H-NMR was recorded on Bruker AVIII 500 MHz Spectrometer and samples were dissolved in CD_3_OD. The IR spectra were recorded using a SHIMADZU IRAffinity-1S, FTIR spectrophotometer, Serial No. A21965100204: dry samples were placed on diamond disk. All electrochemical data were collected at room temperature with a Palmsens4 potentiostat using dimethylsulfoxide (DMSO) as the solvent, with 0.1 M tetrabutylammonium hexafluorophosphate (TBAPF_6_) as the supporting electrolyte. The electrolyte was thoroughly degassed with argon before cyclic voltammograms were collected. A three-electrode configuration was used, consisting of a glassy carbon button working electrode (area = 0.071 cm^2^), Pt wire as the counter electrode and an Ag/AgNO_3_ pseudo reference electrode. Potentials are then reported versus the ferrocenium/ferrocene couple, the position of which was found by spiking samples with ferrocene. A scan rate of 100 mV s^−1^ was used for cyclic voltammetry. The absorption spectra of the samples were recorded at room temperature using a Cary 60 instrument in 1 cm pathlength cuvettes and in DMSO as the solvent. The concentration of all samples in the reported spectra were 0.6 mg ml^−1^.

## Result and discussion

### Structural analysis

**Fig. 2:**
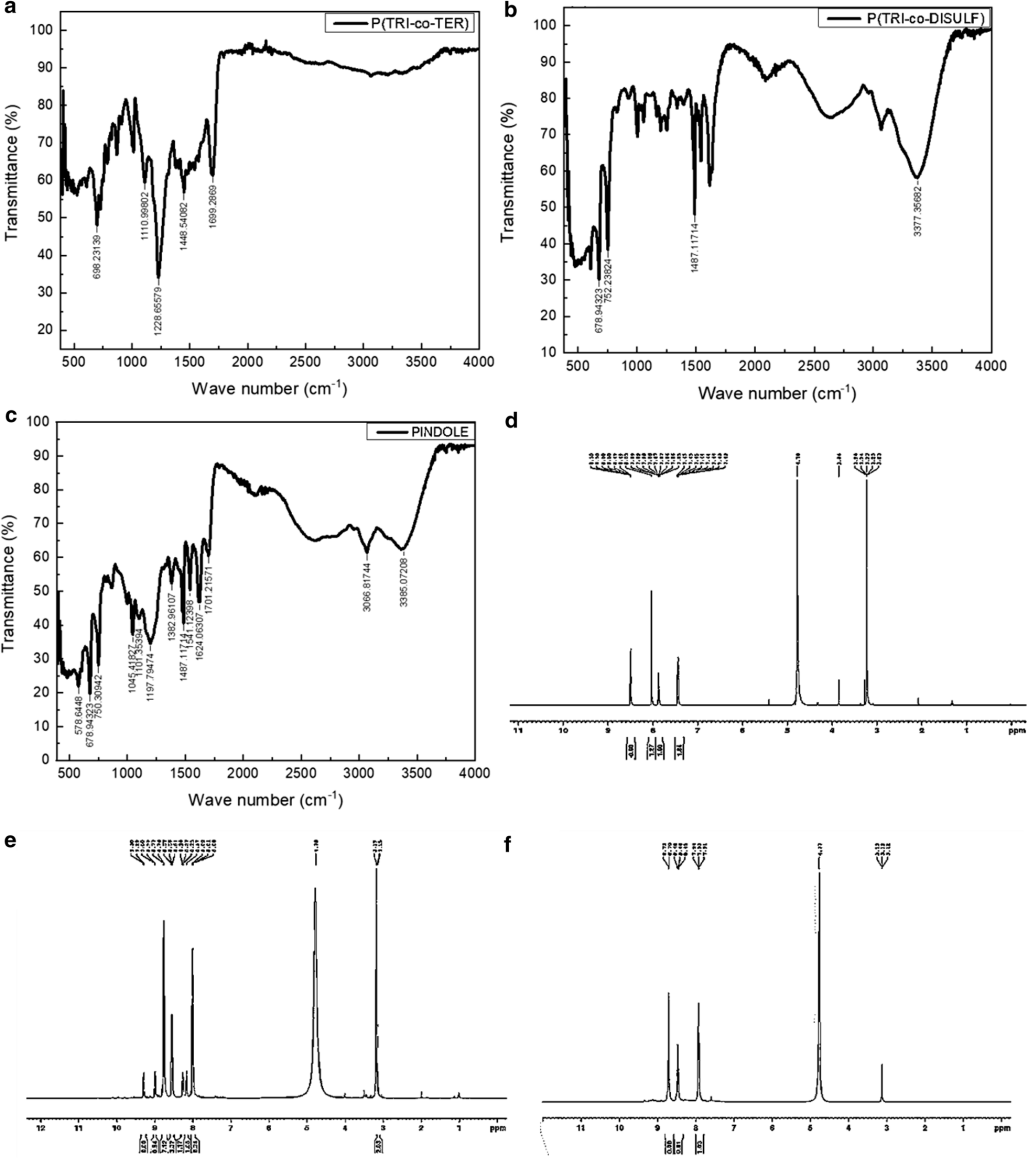
**a**–**c** FTIR spectra and **d**–**f** ^1^H-NMR spectra for the P(TRI-co-TER), P(TRI-co-DISULF) and PINDOLE polymers

Fourier transformation infrared (FTIR) and nuclear magnetic resonance (NMR) spectroscopy were utilized to perform the structural analysis of the three synthesized polymers. FTIR spectra can be used to reveal the molecular structure and molecular environment due to vibrational modes [21]. Figure 2a–c shows the FTIR spectra for the three synthesized polymers, denoted by P(TRI-co-TER), P(TRI-co-DISULF), and PINDOLE, while their main IR characteristic modes are shown in Table 2. The absorption bands around 3000 to 3400 cm^−1^ are assigned to C–H stretching for the aromatic rings and N–H stretching for normal vibration of the pyrrole rings, respectively [22, 23]. In addition, these bands are broad and weak for P(TRI-co-TER), but sharp and moderate for the P(TRI-co-DISULF) and PINDOLE materials. This could be because the intensity of an absorption band depends on the size of the change in dipole moment associated with the vibration and on the number of bonds responsible for the absorption [24]. Moreover, the formation of cyclic dimers due to the presence of the carboxylic group, which consists of a proton donor and a proton acceptor group, can lead to the presence of an intermolecular hydrogen bond of the carboxylic acid with pyridine and an intramolecular hydrogen bond of the proton donor N–H with oxygen [21]. The band at 1700 cm^−1^ was attributed to the stretching vibration of the carboxylic group C=O [24]. This band was observed for both P(TRI-co-TER) and PINDOLE, whereas sulfoxide functional groups (S=O) were perceived at 1371 and 1325 cm^−1^ for P(TRI-co-DISULF) [24]. The out-of-plane bending mode for C–H in the spectral region from 400 to 1000 cm^−1^ could be due to the benzene ring because of the polymerization process [25]. Mono substitution bands were observed for all investigated polymers, whereas para substitutions were perceived for P(TRI-co-DISULF) and PINDOLE. ^1^H-NMR spectra for all synthesized polymers are shown in Fig. 2d–f and from their spectra it is possible to verify their structure. 5 phenyl protons in triamterene were observed for two polymers, namely P(TRI-co-TER) and P(TRI-co-DISULF), but the protons in P(TRI-co-DISULF) experienced a downfield shift due to the electronegativity effect and resonance [24, 26]. In addition, 4, 8 aromatic protons in the terephthalate and diphenyl group at chemical shift $\delta 8 {\text{ppm}}$ were perceived for P(TRI-co-TER) and P(TRI-co-DISULF), respectively [26]. Chemical shifts at peaks of $\delta 8.56$ and $\delta 8.46$ are assigned to NH for both P(TRI-co-DISULF) and PINDOLE, respectively: the up-field shift for NH is due to the electronegative effect [24].

**Table 2:** Assignment of FTIR spectra for the three synthesized polymers

Polymer	Wave number (cm^−1^)	Assignment
P(TRI-co-TER)	1699	C=O stretching for carboxylic acid [24]
	1448	N–H and C–H in plain bending, C–C stretching for normal vibration of pyrrole ring [22]
	1228	C–N stretching at benzene ring [27]
	1110	${\text{NH}}^{ + }$ stretching between quinones and benzene ring [27]
	698	C–H out of plane bending at aromatic ring [24]
P(TRI-co-DISULF)	3377	N–H stretching for normal vibration of pyrrole ring [22]
	3066	C–H stretching at aromatic ring [23]
	1624	C=N stretching in quinones ring [27]
	1541	C=C stretching in quinones ring [25]
	1487	N–H and C–H in plain bending, C–C stretching [28]
	1371, 1325	S=O stretching [24]
	752	C–H, and N–H out of plain bending [28]
	678	C–H, and N–H out of plain bending [28]
PINDOLE	3385	N–H stretching for normal vibration of pyrrole ring [22]
	3066	C–H stretching at aromatic ring [23]
	1701	C=O stretching for carboxylic acid [24]
	1624	C=N stretching in quinones ring [25]
	1541	C=C stretching in quinones ring [25]
	1487	N–H and C–H in plain bending, C–C stretching [28]
	1382	N–H and C–H in plain bending, C–C stretching [28]
	1197	C–H in plain bending [22]
	1101	${\text{NH}}^{ + }$ stretching between quinones and benzene ring [27]
	1045	C–O–C stretching [24]
	750	C–H, and N–H out of plain bending [28]
	678	C–H, and N–H out of plain bending [28]
	578	C–H, and N–H out of plain bending [28]

### Photophysical and electrochemical properties

**Fig. 3:**
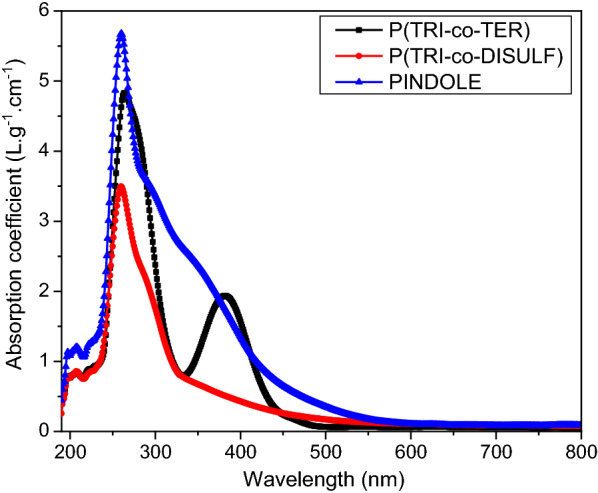
Absorption coefficient spectra for the three synthesized polymers

UV–VIS absorption spectroscopy was used to evaluate the photophysical properties of the three newly synthesized polymers. The polymer solution was prepared by dissolving 0.6 mg of each polymer in 1 ml of dimethyl sulfoxide (DMSO). It is known from literature that the absorption bands in the UV region can be ascribed to the $\pi - \pi^{*}$ and $n - \pi^{*}$ transitions of delocalized excitons in the polymer chain, whereas the absorption bands in the visible range are assigned to intramolecular charge transfer (ICT) between electron-rich moieties and electron-deficient moieties in the main chain [14–17, 20]. The absorption coefficient spectra (Fig. 3) of the polymers were determined using the following equation [29]:

1 $$\alpha = \frac{2.303A}{t}$$

where *t* is the thickness of the cuvette (10 mm) and A is the absorbance. All three polymers exhibited a sharp absorption band in the UV region which extended to the visible region. The absorption band for PINDOLE was prolonged until 470 nm and the absorption band for P(TRI-co-TER) continued until 440 nm, whereas the absorption band for P(TRI-co-DISULF) continued till 400 nm. These indicate that the delocalized excitons’ transition from $\pi - \pi^{*}$ and $n - \pi^{*}$ take place in the polymer backbones for the polymers, whereas the differences of prolonged band in the visible region for the polymers is due to the degree of intramolecular charge transfer (ICT), which is related to the transition of excitons between benzenoid and quinoid rings [30]. Comparably, the absorption edge of our polymers was found to be in the range from 500 to 600 nm, while those for the PID2, BTI-IDT-BTI and PCBM are reported to be at about 580, 670 and 450 nm, respectively [31, 32]. The low absorption coefficients of our polymer solutions compared to that of solid films is a consequence of their dilution in the solvents used [33]. Hence, the absorption coefficients of solid films made from these polymers after removal of the solvent would be higher, and could be high enough for potential applications since the intermolecular pi–pi bonding interactions would also be greatly enhanced.

#### Optical energy gap and transition types

In optoelectronic applications, it is imperative to have the measurement of the optical energy gap and the type of optical transitions in the conjugated polymers when considering the potential application of the polymers. From the absorption spectra, it is possible to find the optical energy gap and optical transition by using Tauc’s equation [34, 35]. Furthermore, the absorption edge from the absorption spectrum has been used to determine the optical energy gap, thereby measuring $\lambda_{onset}$ as follows [36–38]:

2 $$E_{g} = \frac{1242}{{\lambda_{onset} }}$$

**Fig. 4:**
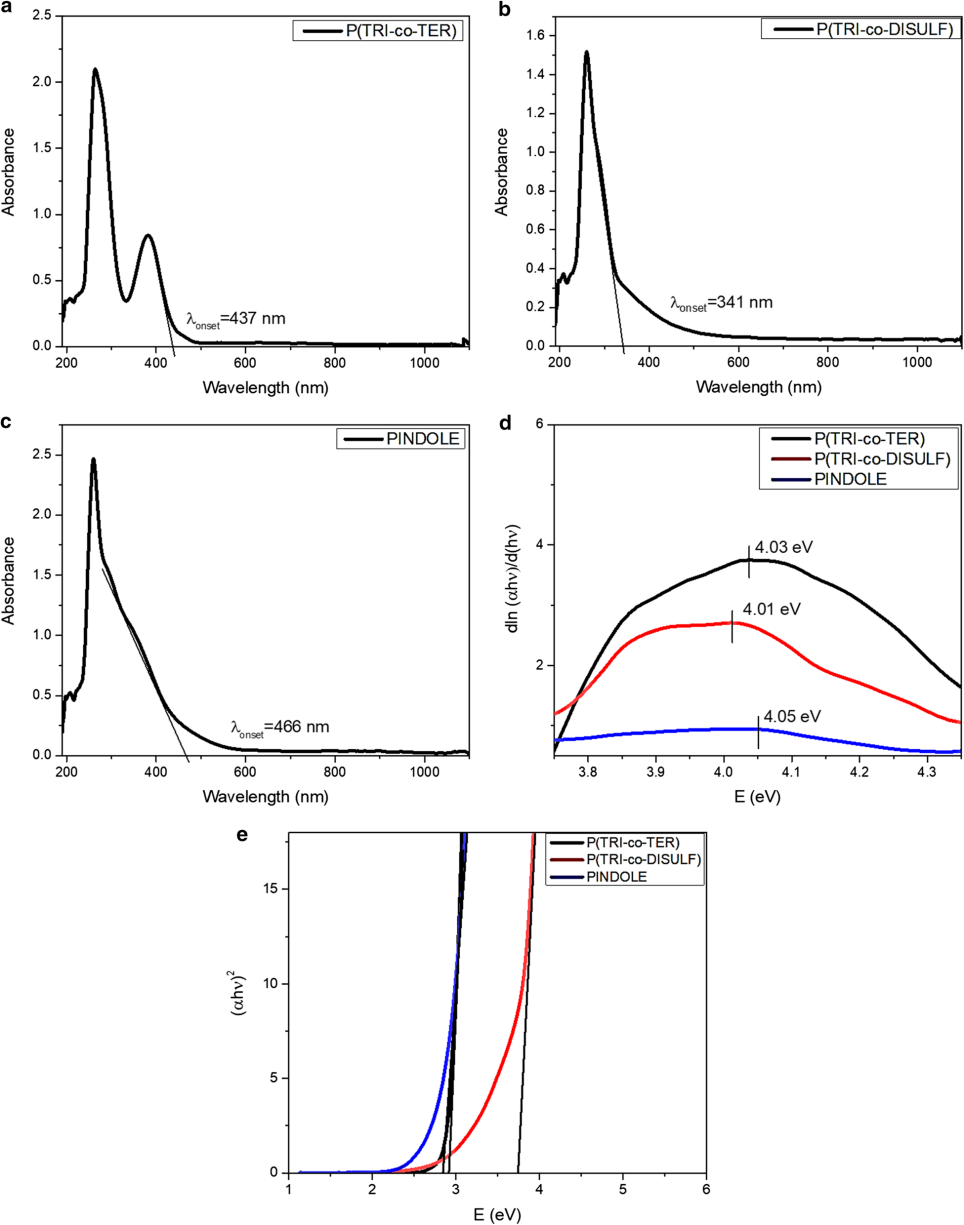
**a**–**c** Absorbance spectra for all synthesized polymers, **d** plot of $dln\left( {\alpha h\nu } \right)/dh\nu$ versus $h\nu$ for all synthesized polymers, and **e** plot of $\left( {\alpha h\nu } \right)^{2}$ versus $E$ for all synthesized polymers

However, Tauc’s equations can be assigned directly to ascribe the nature of the transition despite the measuring of optical energy gap [39], that is by taking the natural logarithm and derivation of Eq. 3,

3 $$\alpha h\nu = \alpha_{o} \left( {h\nu - E_{g} } \right)^{n}$$

4 $$\frac{{d\ln \left( {\alpha h\nu } \right)}}{{d\left( {h\nu } \right)}} = \frac{n}{{h\nu - E_{g} }}$$

where $E_{g}$ is the energy gap, $\alpha_{o}$ is an energy-independent constant, *h* is Planck’s constant, ν is the frequency of the incident wave, and the value of *n* defines the type and nature of the transitions [40]. If the value of $n = 2$, the transition is an indirectly allowed transition, *n* = 3 for indirectly forbidden transitions, *n* = 1/2 for directly allowed transitions and *n* = 3/2 for directly forbidden transitions. Figure 4a–c shows the absorption onset of the polymers and their equivalent optical energy gaps, which were calculated from $\lambda_{onset}$ (Eq. 2) and are listed in Table 3. The plots of $\frac{{d\ln \left( {\alpha h\nu } \right)}}{{d\left( {h\nu } \right)}}$ versus $h\nu$ for all samples are shown in Fig. 4d and the approximate value of $h\nu = E_{g}$ was taken at the peak value. Hence, the estimated value of $E_{g}$ was employed for plotting $\ln \left( {\alpha h\nu } \right)$ versus $\ln \left( {h\nu - E_{g} } \right)$ and the value of *n* was determined from the slope of the curves and was found to be $\frac{1}{2}$, which shows the occurrence of a directly allowed transition between the intermolecular energy bands of the polymers. Then, the accurate values of the energy gaps were determined by Tauc’s equation by plotting $\left( {\alpha h\nu } \right)^{2}$ as a function of $\left( {h\nu } \right)$ and taking the extrapolation of the linear portion at $\left( {\alpha h\nu } \right)^{2} = 0$. The positions of the energy gaps are depicted in Fig. 4e for all the polymers. Also, the determined values of $E_{g}$ are shown in Table 3.

**Table 3:** Determined energy gap for synthesized polymers from absorbance data

Polymer	$E_{g}^{opt.}$	
	$E_{g}^{{\lambda_{onset.} }}$	$E_{g}^{Tauc^{\prime}s.}$
P(TRI-co-TER)	2.84 eV	2.92 eV
P(TRI-co-DISULF)	3.63 eV	3.75 eV
PINDOLE	2.66 eV	2.85 eV

#### Electrochemical properties

There are several parameters that should be considered in designing and optimizing organic photovoltaic devices which include charge transfer and charge collection at the active medium and electrodes. In this respect, electrochemical study provides information regarding the position of the HOMO and LUMO levels of organic materials prior to device fabrication. Cyclic voltammetry (CV) is a good method to estimate energy levels from the oxidation and reduction potentials for the relevant materials. The oxidation and reduction potentials are inferred from the onset potential, which is defined as the potential where holes or electrons are initially injected into the HOMO and LUMO levels, respectively, and the rise of anodic or cathodic current becomes obvious [41]. In order to estimate the position of the HOMO and LUMO levels, first, optical energy gaps were estimated from Tauc’s equation (Section “Optical energy gap and transition types”). Second, the LUMO and HOMO levels were calculated from the observable reduction and oxidation potentials from CV measurements for all the polymers. Then, the HOMO and LUMO levels were estimated from the relation below using ferrocene as the reference couple [19, 42, 43]:

5 $$E_{HOMO} = - \left( {E_{{\left( {onset,ox vs.Fc^{ + } /Fc} \right)}} + 5.39} \right)\left( {eV} \right)$$

6 $$E_{LUMO} = - \left( {E_{{\left( {onset,red vs.Fc^{ + } /Fc} \right)}} + 5.39} \right)\left( {eV} \right)$$

7 $$E_{g}^{Tauc} = E_{HOMO} - E_{LUMO}$$

**Fig. 5:**
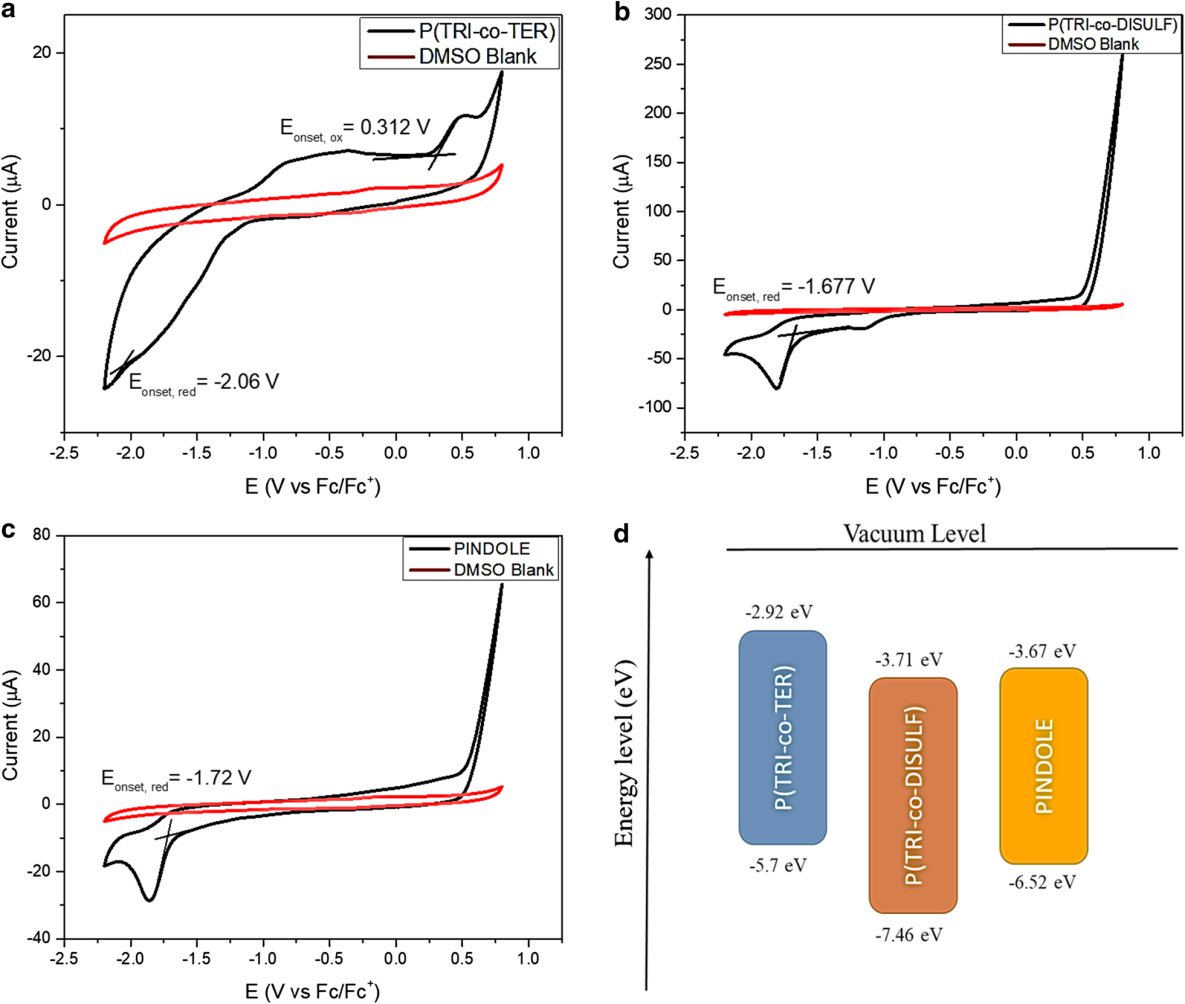
**a**–**c** The Cyclic Voltammetry (CV) spectra for all synthesized polymers, and **d** Energy level diagram for all synthesized polymers. The small redox wave at about − 1.1 V *vs.* Fc^+^/Fc in the CV of P(TRI-co.-DISULF) corresponds to the reduction of adventitious trace oxygen in the DMSO solvent as previously reported [46]. The irreversible oxidative processes observed in the CVs in **a** and **b** are likely due to the oxidation of adventitious water in the DMSO solvent

Illustrative CVs of the three polymers, versus Fc/Fc^+^, are presented in Fig. 5a–c, while the corresponding electrochemical parameters compared to some donor and acceptor materials reported in literature are shown in Table 4. Moreover, a complete diagram of the HOMO and LUMO energy levels of these polymers is shown in Fig. 5d. The HOMO level is influenced by the type of substituents (whether electron withdrawing or electron donating species [19]) and it can be seen that P(TRI-co-TER) experienced a high HOMO level compared to that of the other two polymers. This could be due to the presence of the indole N–H group, whereas P(TRI-co-DISULF) and PINDOLE have deeper HOMO levels, due to the double bonds’ conjugation. Furthermore, the deeper HOMO level is useful for devices with high open circuit voltage (*Voc*) and stability in air [14]. The LUMO level of P(TRI-co-DISULF) is deeper compared to that of P(TRI-co-TER) and PINDOLE, which could be related to high molecular weight and electron conjugation in P(TRI-co-DISULF) [19]. In addition, P(TRI-co-TER) has relatively similar molecular energy levels to that of P3HT, and also the LUMO level of P(TRI-co-TER) is 2.92 eV (calculated from *E*_*ox*_ and *E*_*g*_ because of the weak observation of *E*_red_ on the CV plot), which is about 0.4 V or more greater than the LUMO level of PCBM. This suggests that the offset energy between them is necessary to produce effective cascade charge transfer at the donor–acceptor interface whenever P(TRI-co-TER) is used for cascade charge transfer purposes [44]. In contrast, PINDOLE has rather similar energy levels to that of PCBM, making it a potential candidate to be used as an electron acceptor in polymer photovoltaic devices. Moreover, based on the energy level diagram of the three polymers and their absorption spectra response, the polymers’ viability can be further explored [44]. For the possible application of organic solar cells, it is imperative to have photoinduced charge transfer taking place between the donor and acceptor components within the active layer [45]. In our polymers, the photoinduced charge transfer, which is resulted from the absorption of light energy, can be expressed by the transport of electrons from the active moieties of P(TRI-co-TER) or P(TRI-co-DISULF) to the PINDOLE polymer matrix in the system of donor–acceptor heterojunctions.

**Table 4:** Electrochemical and optical data for all synthesized polymers

Polymer	$E_{onset, ox}$(V)	$E_{onset, red}$(V)	$E_{HOMO}$(eV)	$E_{LUMO}$(eV)	$E_{g}^{opt.}$(eV)	References
P(TRI-co-TER)	0.312	− 1.24	− 5.70	− 2.78	2.92	This work
P(TRI-co-DISULF)	NA	− 1.68	− 7.46	− 3.71	3.75	This work
PINDOLE	NA	− 1.72	− 6.52	− 3.67	2.85	This work
PID2	NA	NA	− 5.52	− 3.50	2.02	[32]
BTI-IDT-BTI	0.50	− 1.60	− 5.30	− 3.20	2.10	[31]
PCBM	1.5	− 0.98	− 6.18	− 3.7	2.48	[47]

#### Optical constants

**Fig. 6:**
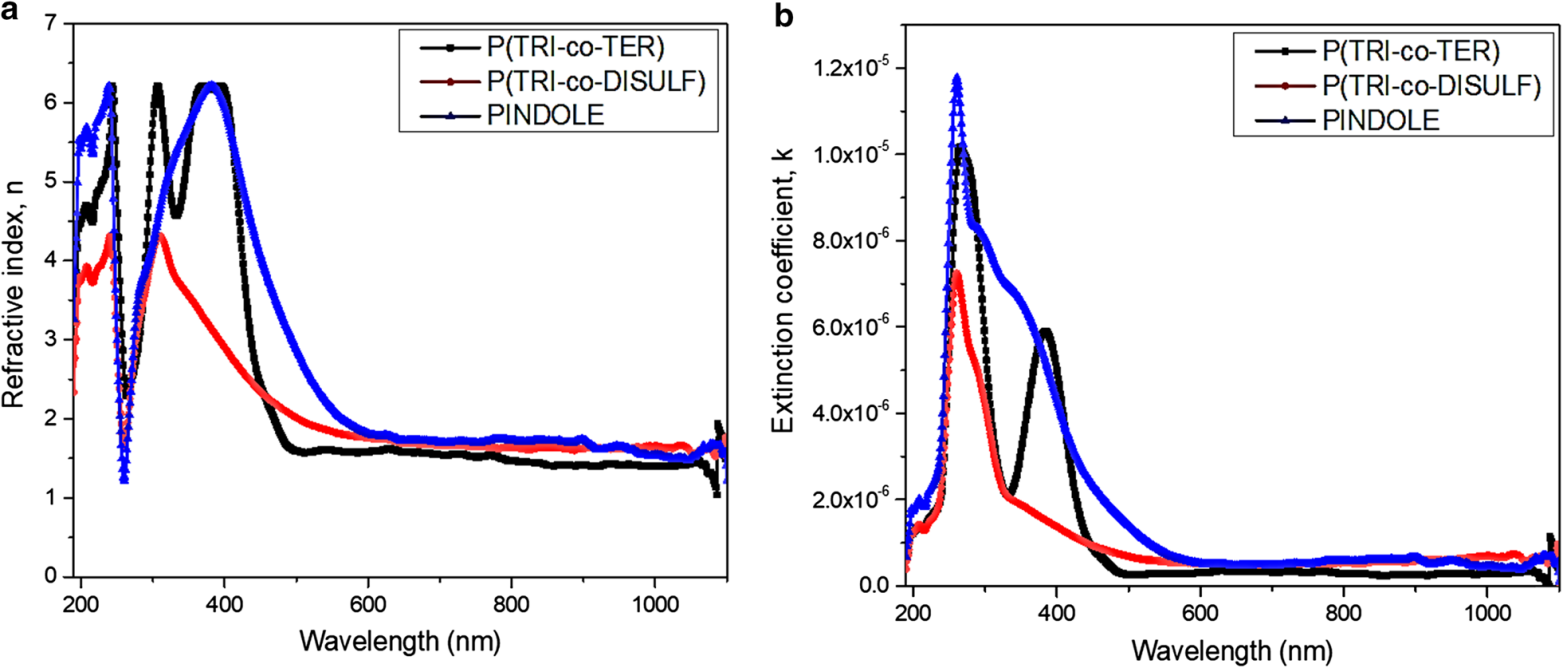
**a** Refractive index and **b** extinction coefficient spectra for the synthesized polymers

Optical constants such as refractive index and extinction coefficient and their derivative parameters like dielectric constant and optical conductivity should be considered before employing the materials in photovoltaic devices. How the electromagnetic wave spreads throughout the materials and the change of the speed inside the material with respect to the vacuum is revealed by studying the refractive index. Moreover, it is a complex variable and the imaginary part indicates the amount of energy lost due to the medium and it is called the extinction coefficient. The absorbance data were used to calculate both refractive index (n) and extinction coefficient (k) using Eqs. 8 and 9 [48].

8 $$n = \frac{{ - 2\left( {R + 1} \right) \pm \sqrt {4k^{2} R^{2} + 16R - 4k^{2} } }}{{2\left( {R - 1} \right)}}$$

9 $$k = \frac{\alpha \lambda }{{4\pi }}$$

where $\alpha$ is the absorption coefficient and R is the reflectance. They were calculated using Eq. 4 and the following equation:

$${\text{R}} = 1 - {\text{T}} - {\text{A}},$$

where A is absorbance and T is transmittance and estimated from ${\text{T}} = 10^{{ - {\text{A}}}}$. Figure 6a, b shows the variation of refractive index and extinction coefficient, respectively, as a function of wavelength from 200 to 1100 nm. Results show that the P(TRI-co-TER) and PINDOLE have a wide dispersion region between 300 and 500 nm and 300 nm to 450 nm, respectively, whereas P(TRI-co-DISULF) had a narrow dispersion region between 300 and 400 nm. The plateau region of the refractive index was observed at high wavelength and the extrapolation of the curve to the y-axis was used to the measure the static value of refractive index, as presented in Table 5. It is worth noticing that P(TRI-co-TER) has the lowest refractive index value while PINDOLE presented the highest value. These are due to polarization of the molecules in the polymers with the electromagnetic wave, thus the broadening of the peaks and the static value of (*n*) resulting from the polar nature of the polymers which is described by the resonance effect between electron polarization and incident light [49, 50]. Furthermore, the extinction coefficient (*k*) designates the loss of the incident photon due to scattering and absorption within the medium. Noticeably, the variation of (*k*) is almost comparable to the corresponding absorption coefficient (Eq. 9) [49]. All samples show a sharp absorption region in the UV and extending into the visible region to different extents.

**Table 5:** The optoelectronic parameters estimated for the synthesized polymers

Polymer	*n*	$\varepsilon_{r}$	$\sigma_{r} \times 10^{ - 4} {\text{S}}\,{\text{cm}}^{ - 1}$
P(TRI-co-TER)	1.56	2.44	2.43
P(TRI-co-DISULF)	1.62	2.63	3.31
PINDOLE	1.68	2.91	4.13

**Fig. 7:**
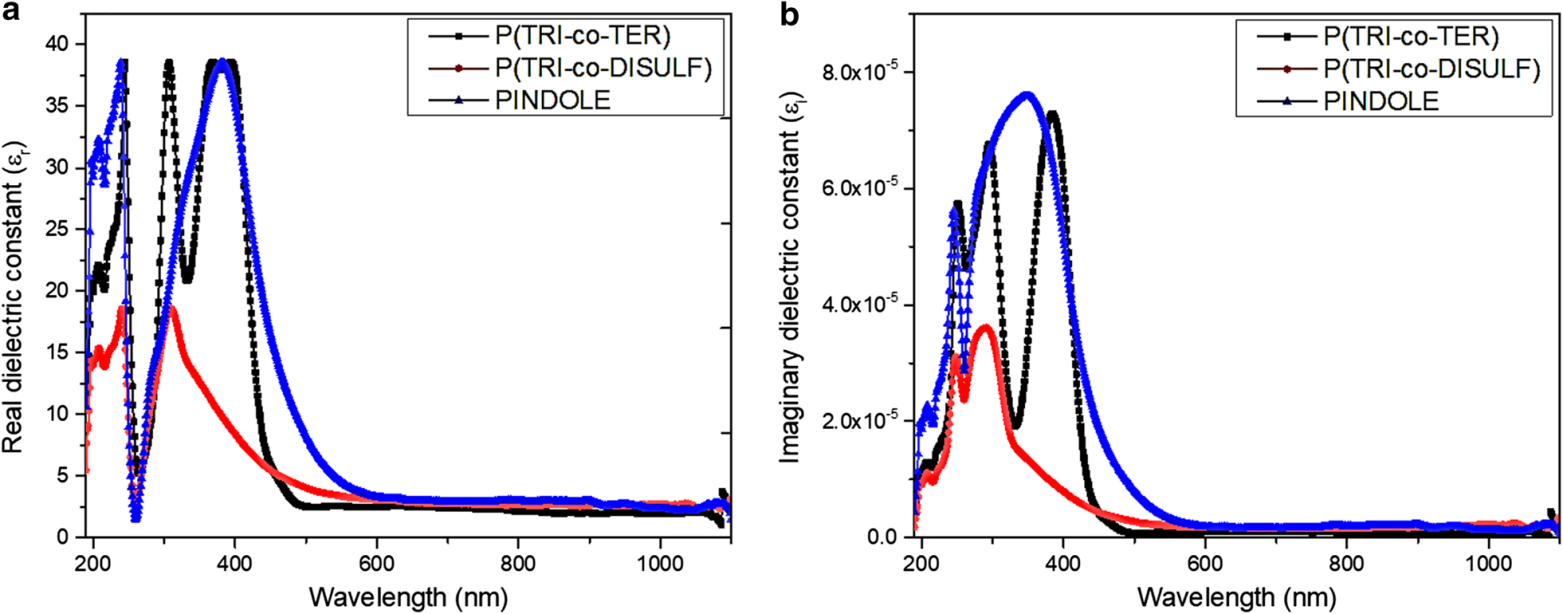
Dielectric constant spectra for all synthesized polymers **a** Real part, and **b** Imaginary part

The optical dielectric constant ($\varepsilon$) is a frequency dependent parameter and shows the electronic response to the incident photon in the material. Meanwhile, the dielectric constant is a complex function and its real part is assigned to polarization upon the impact of an electromagnetic field whereas the imaginary part illustrates the optical loss and is described by the following equations [51]

10 $$\varepsilon = \varepsilon_{1} + i\varepsilon_{2}$$

11 $$\varepsilon_{1} = n^{2} - k^{2}$$

12 $$\varepsilon_{2} = 2nk$$

13 $$\tan \delta = \frac{{\varepsilon_{2} }}{{\varepsilon_{1} }}$$

where $\varepsilon_{1}$ represents the real part and $\varepsilon_{2}$ represents the imaginary part of the dielectric constant. Figure 7a, b shows the fluctuation of optical dielectric constant with respect to the wavelength from 200 to 1100 nm.

**Fig. 8:**
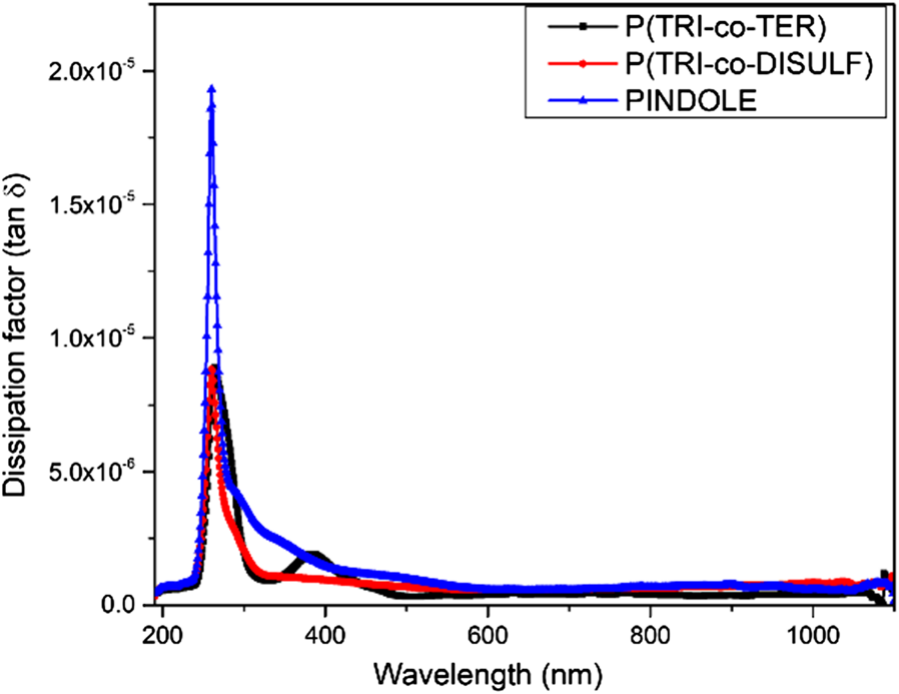
Dielectric lost tangent (dissipation factor) spectra for all synthesized polymers

Noticeably, the real part of the spectrum of the optical dielectric constant reflects the refractive index because of the small value of *k*, while the imaginary part is essentially based on the absorption coefficient (see Eqs. 9, 11 and 12). The observed peaks for P(TRI-co-TER) and PINDOLE in the real part of the spectrum are positioned in the visible region, while in the imaginary part there is a broad peak from the UV to the visible region for PINDOLE and doublet peaks for P(TRI-co-TER) positioned at 295 and 385 nm. However, P(TRI-co-DISULF) shows narrow peaks at 300 and 285 nm for real and imaginary part of the dielectric constant, respectively. The value of the real dielectric constant was measured at high wavelength by taking the extrapolation of the continued part to the y-axis, as shown in Table 5. It was seen that PINDOLE presented the maximum value, which indicated the presence of more interaction between incident photon and electrons in this polymer compared to that of other polymers [52]. In addition, the dissipation factor ($\tan \delta$) was calculated from Eq. 13, which relates to the rate of absorption [34]. The dissipation factors spectra for all samples are shown in Fig. 8. From the characteristic curve of the dissipation factor, it can be noticed that PINDOLE experiences the highest absorption rate in the UV region (and extended to visible region), whereas P(TRI-co-TER) and P(TRI-co-DISULF) show a moderate absorption rate starting in the UV region and extending to the visible region, with a maximum wavelength centered in the UV region.

**Fig. 9:**
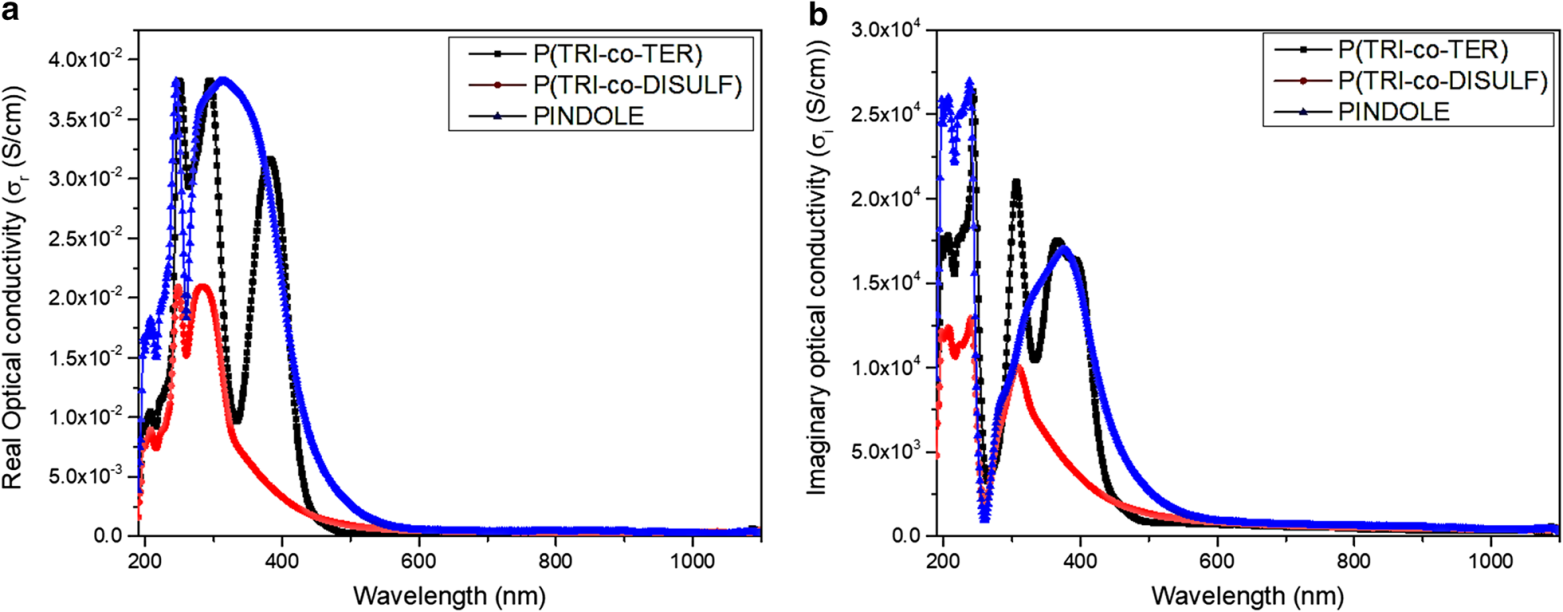
Optical conductivity spectra for all synthesized polymers **a** Real part, and **b** Imaginary part

The essential parameter which can be used to describe the electron response to the absorbed electromagnetic wave is optical conductivity. Since the optical conductivity is derived from the optical dielectric constant, it is a complex variable and the following equations define both parts of the optical conductivity [53]:

14 $$\sigma^{*} = \sigma_{r} + i\sigma_{i}$$

15 $$\sigma_{r} = \omega \varepsilon_{2} \varepsilon_{o}$$

16 $$\sigma_{i} = \omega \varepsilon_{1} \varepsilon_{o}$$

where $\omega$ is the angular frequency, $\varepsilon_{o}$ is the permittivity of free space, $\sigma_{r}$ and $\sigma_{i}$ are the real and imaginary parts of complex optical conductivity, respectively. From the above equations, it can be understood that the real optical conductivity is linked to the imaginary optical dielectric constant and therefore depends on the absorption coefficient. Although, the imaginary optical conductivity is connected to the real optical dielectric constant, which describes the polarization due to interaction between photons and electrons. The optical conductivity spectra are shown in Fig. 9a, b and the values of the real optical conductivity measured from the extrapolation of the prolonged part of the spectrum at high wavelength are presented in Table 5. The transport response of electrons in the polymer chain is directly related to the value of the real optical conductivity, which is defined by the energy of the absorbed photon [54]. It worth mentioning that PINDOLE shows the maximum value, whereas P(TRI-co-TER) shows the minimum value. It is evidenced from their spectra that the molecular interaction between moieties of the polymer chains take place at high energy levels.

## Conclusions

In conclusion, two new electron rich and one electron deficient polymers have been synthesized in one step reactions and characterized. The three polymers showed relatively high optical band gaps with deep HOMO levels, making them strong absorbers of photons in the UV region, with absorption extending into the visible region. Furthermore, the synthesized polymer named PINDOLE has highest optoelectronic constants compared to the other polymers. Results suggested that the newly synthesized polymers might be used as donor and acceptor materials in bulk heterojunction structures (BHJ), making them viable for semi-transparent photovoltaics applications. Moreover, the newly synthesized donor and acceptors show good performance relative to P3HT and fullerenes due to the close match of their HOMO and LUMO levels, respectively.

## Data Availability

The data and material are available within the manuscript.
